# Patient Perspective of Cognitive Symptoms in Major Depressive Disorder: Retrospective Database and Prospective Survey Analyses

**DOI:** 10.2196/11167

**Published:** 2019-05-16

**Authors:** Emil Chiauzzi, Jennifer Drahos, Sara Sarkey, Christopher Curran, Victor Wang, Dapo Tomori

**Affiliations:** 1 PatientsLikeMe Cambridge, MA United States; 2 Takeda Pharmaceuticals Cambridge, MA United States; 3 Takeda Pharmaceuticals Deerfield, IL United States

**Keywords:** cognition, cognitive function, depression, major depressive disorder, patient-centered care, patient preference, relapse, remission, symptoms

## Abstract

**Background:**

Major depressive disorder (MDD) is a common and burdensome condition. The clinical understanding of MDD is shaped by current research, which lacks insight into the patient perspective.

**Objective:**

This two-part study aimed to generate data from PatientsLikeMe, an online patient network, on the perception of cognitive symptoms and their prioritization in MDD.

**Methods:**

A retrospective data analysis (study 1) was used to analyze data from the PatientsLikeMe community with self-reported MDD. Information on patient demographics, comorbidities, self-rated severity of MDD, treatment effectiveness, and specific symptoms of MDD was analyzed. A prospective electronic survey (study 2) was emailed to longstanding and recently active members of the PatientsLikeMe MDD community. Study 1 analysis informed the objectives of the study 2 survey, which were to determine symptom perception and prioritization, cognitive symptoms of MDD, residual symptoms, and medication effectiveness.

**Results:**

In study 1 (N=17,166), cognitive symptoms were frequently reported, including “severe” difficulty in concentrating (28%). Difficulty in concentrating was reported even among patients with no/mild depression (80%) and those who considered their treatment successful (17%). In study 2 (N=2525), 23% (118/508) of patients cited cognitive symptoms as a treatment priority. Cognitive symptoms correlated with depression severity, including difficulty in making decisions, concentrating, and thinking clearly (r_s_=0.32, 0.36, and 0.34, respectively). Cognitive symptoms interfered with meaningful relationships and daily life tasks and had a profound impact on patients’ ability to work and recover from depression.

**Conclusions:**

Patients acknowledge that cognitive dysfunction in MDD limits their ability to recover fully and return to a normal level of social and occupational functioning. Further clinical understanding and characterization of MDD for symptom prioritization and relapse risk due to residual cognitive impairment are required to help patients return to normal cognitive function and aid their overall recovery.

## Introduction

Major depressive disorder (MDD) is a common disorder, with the lifetime prevalence estimated to be just over 16% in the United States [1]. MDD is also a burdensome condition: It is the third-leading global cause of disability according to the World Health Organization [2]. The epidemiology of MDD is affected by a number of factors. In addition, its lifetime prevalence is higher in high-income (14.6%) than in low-to-middle income (11.1%) countries [3]. In addition, women typically have a much higher risk of MDD than men (odds ratio, 1.6-2.7 in developed countries), and a substantial proportion of patients receiving treatment for MDD have chronic recurrent illnesses alongside their depressive symptoms [3].

Understanding patients’ views on the burden and trajectory of symptoms and the extent to which different symptoms affect daily life is a necessary step toward addressing the most troublesome symptoms of MDD in ways that improve patients’ abilities to meet their personal and occupational goals [4]. Ultimately, the aim of obtaining patient insights into MDD is not only to improve treatment and recovery but also to ensure optimal treatment of MDD in order to benefit society.

Mood disturbance, problems with concentration/attention, and physical symptoms are core criteria used to diagnose MDD [5]. Impairment in cognitive function, in particular, those related to executive function, concentration, learning, and memory, is well recognized in MDD [6,7]; indeed, cognitive symptoms appear to be a core feature of the disorder [8] and a key mediator of functional impairment [9,10]. Even after other depressive symptoms are abated, unresolved cognitive deficits can lead to continued social, functional, and occupational disabilities [11]. In addition, persistent cognitive symptoms have been associated with an increased risk of relapse [12]. At present, there is no consensus among clinicians on the most-appropriate tools for assessing cognitive function in MDD. In addition, current cognitive evaluation methods lack the patient perspective and require more formal neuropsychological assessment [10,13,14]. Patients’ views on the significance and effects of cognitive symptoms are therefore important in order to fully understand the subjective experience of MDD and the role of these symptoms in the disorder.

A lack of shared decision‑making behaviors has been highlighted in depression care [15], and many health care providers (HCPs) find the structured assessment of depression burdensome [16]. Collectively, these issues may limit the ability of HCPs to fully account for the patient experience of MDD and tailor care to the needs of individuals. Considering the potentially different perspectives of patient and provider assessments of disease experience and burden in MDD (ie, disease severity, disease improvement, and symptom prioritization) [4,17-19], it is important to evaluate patients’ perspectives on their own disorder. This approach emphasizes the importance of gaining patient insights on particular symptoms of the disorder to optimize patient care. To further understand patients’ perspectives, we performed Patient Insights and Voice on Major Depressive Disorder Treatment and Symptom Perception (PIVOT) studies using the PatientsLikeMe [20] online patient research network.

PatientsLikeMe is an online patient research network that provides a forum for sharing real-world health experiences to improve patient outcomes, allows patients to track their own conditions, and gathers crowdsourced data that can be used for disease research. PatientsLikeMe has approximately 30,000 patients with self-reported major depression and is a source of both qualitative and quantitative patient-reported data and perspectives about disease symptoms, treatments, and comorbidities. Patient engagement and data donations enable PatientsLikeMe researchers to invite members to participate in research projects that may be valuable to the patients based on their self-reported data. Here, we report the results of two PIVOT studies that examine data from subsets of the PatientsLikeMe MDD community. These two studies were designed to analyze existing platform data on patients’ perceptions of MDD symptoms, with a focus on cognitive symptoms and medication effectiveness (study 1: retrospective database analysis) and ways patients understand and prioritize symptoms of depression (study 2: prospective survey). Study 1 analyzed a wide array of data, which were used to determine the exploratory research questions to be addressed in study 2 and enable construction of the survey. Thus, this report primarily focuses on the outcomes of study 2.

## Methods

### Ethical Approval

All data were provided voluntarily by patients, and PatientsLikeMe was granted exemption from ethical approval by the New England Institutional Review Board (exemption number 15-349; September 24, 2015).

### Study 1: Retrospective Database Analysis

#### Data Collection

Members of PatientsLikeMe provide demographic information such as age, race, educational status, health insurance coverage, and location to help match demographically similar patients. They also identify primary and comorbid medical conditions from among more than 2500 conditions in the system or enter a new condition. Patients can complete a list of their symptoms and rate the current severity of symptoms (none/mild/moderate/severe), both of which can be updated over time. In addition, patients are invited to rate the effectiveness of treatments, including prescription and over-the-counter medications. These treatment evaluations also include treatment burden, adverse effects, adherence, and cost. Furthermore, the PatientsLikeMe platform database collects free-text entries, in which members provide qualitative, narrative information. Individuals were not required to complete all fields in the PatientsLikeMe system for their data to be included in this database analysis.

In the retrospective database analysis, data were examined from the entire PatientsLikeMe community of patients who reported having MDD (“the total MDD community”). This community comprised 17,166 individuals as of June 1, 2015. Patients who selected MDD among their conditions in the PatientsLikeMe system were prompted to provide feedback on specific symptoms, including depressed mood, fatigue, insomnia, inability to experience pleasure (anhedonia), low self-esteem, lack of motivation, and problems with concentration.

### Data Analysis

All patients were included in the analysis irrespective of whether they had missing data. In cases where data were incomplete, the total frequency of respondents (n) was used and the missing data were not imputed.

Demographics, clinical characteristics, and treatment covariates of interest are presented using descriptive statistics. Continuous variables are described using the number of observations, including mean and SD, for nonnormally distributed data. Categorical variables were described using frequency and percentage (n, %) or relative percentage of values in each category. Data were analyzed using STATA, version 13.0 (StataCorp LP, College Station, TX) and RStudio, version 0.99.902 (RStudio, Inc, Boston, MA).

### Study 2: Prospective Survey Questionnaire

#### Survey Design

The custom survey was developed by all authors using information obtained during the study 1 analysis and was written in a way that was understandable to individuals with an average Flesch–Kincaid [21] reading grade of 7.5. The survey (Multimedia Appendix 1) included items comprising demographic information and patient characteristics (six items); medication history (three items); patient experience of depression in brief (three items); the patient health questionnaire (PHQ-8) depression scale (eight items), which is an adaptation of the clinically used PHQ-9 scale [22] that omits one item relating to suicidal thoughts and is a commonly used and accepted brief screening measure of depression [23]; patient prioritization of depressive symptoms (seven items); patient experience of memory, thinking, and concentration (seven items); patient-reported cognitive function using an adapted version of the perceived deficits questionnaire (PDQ-5), which is a cognitive function severity scale ranging from 0 to 20 (0=low perceived impairment) [24] and has been validated in patients with major depression [25], and using patient perception of cognitive symptoms and depressive relapse (11 items in total); and seeking advice and life adjustments (four items).

Longstanding (n=288; rated depressed mood at least six times during membership and active within the last 30 months) and recently active (n=3112; active within the last 90 days) members of the “total MDD community” were individually invited to participate in the PIVOT survey (total of 3400). Members were invited to participate via private message through the PatientsLikeMe platform. The private message contained research patient information and links to accept or decline the invitation to participate in the study.

**Figure 1:**
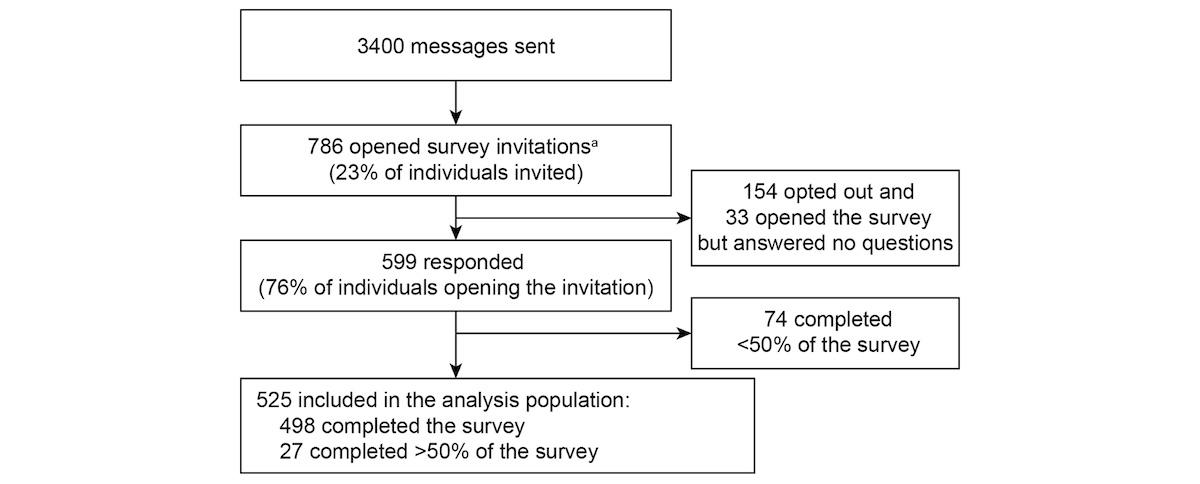
Study 2 patient flow for prospective PIVOT survey in patients with MDD. *Number of unique users who opened one or more survey invitations or reminder emails. PIVOT: Patient Insights and Voice on Major Depressive Disorder Treatment and Symptom Perception.

Patients who accepted the invitation were directed to the electronic survey. To maximize participation, an email reminder was sent to users who did not accept or decline the survey invitation within 3 days. An email reminder was also sent to users who partially completed the survey within 3 days of their last survey data entry. The patient flow is shown in Figure 1. Only those who completed at least half of the survey were included in the analysis sample (N=525). Patients were not compensated for their participation.

#### Data Analysis

Similar to study 1, the total frequency of survey respondents (n) was used, and missing data were not imputed. Data analysis of the prospective survey questionnaire was performed in line with that reported for the retrospective database analysis in study 1. Response rates were calculated using the checklist for reporting results of the internet e-survey criteria [26]. Severity levels on the PHQ-8 depression scale were scored according to Centers for Disease Control and Prevention guidelines (none = 0-4; mild = 5-9; moderate = 10-14; moderately severe = 15-19; and severe = 20-24) [23]. If data were missing for any question, the PHQ-8 score was deemed to be missing. The Spearman rank correlation was used to measure the degree of association between cognitive symptoms and depressive severity. Some demographic information that was not directly requested in the survey was taken from the PatientsLikeMe platform database and included the country of origin and comorbidities. Survey data were reported in cases where both platform and survey data were available.

Open-response questions were reviewed and analyzed for themes. Commonly reported themes were assigned a unique code, and the frequencies of codes were counted. Participants responding with multiple themes per question were counted for each theme reported; thus, the themes were not mutually exclusive by participant and question.

## Results

### Study 1

#### Patients

The “total MDD community” comprised 17,166 individuals. The majority were women (77%); were white (89%); lived in the United States (75%); and had at least some college education, an undergraduate degree, or a professional degree (80%; Table 1). The MDD characteristics of this population are shown in Table 1. Overall, 78% of individuals reported that they had, at some point, received treatment for MDD. In addition, 65% indicated the current severity of their depressed mood as moderate/severe (compared with 25% reporting mild and 10% reporting no depressed mood). These patients reported a wide variety of comorbidities, particularly anxiety disorders and dysthymia.

#### Perception and Impact of Cognitive Symptoms

**Figure 2:**
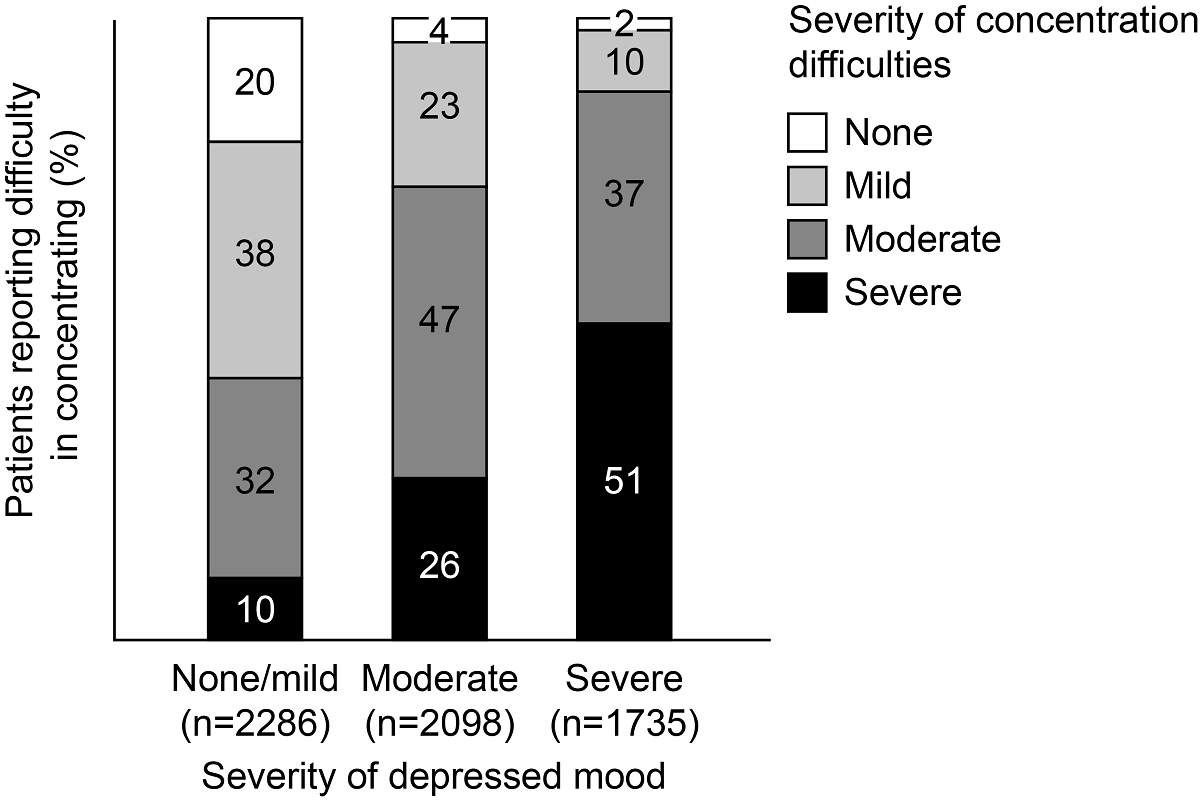
Study 1 self-reported concentration difficulties in patients with major depressive disorder.

Of the total, 38% of patients with data in the system (2255/5998) reported severe lack of motivation and 28% (2889/10,372) reported severe difficulty in concentrating. Among patients with severe depression, those reporting severe difficulty in concentrating were approximately five-fold more in number than those reporting no/mild depressive symptoms (Figure 2). Notably, persistent difficulty in concentration was common in all severities of depression and reported by as many as 80% of patients, even in the absence of depressed mood, or with self-described “mild” depression (Figure 2).

The results of the retrospective database analysis also revealed that across 12 of the most frequently reported medications, only approximately 20% of patient reports (n=3095) indicated major effectiveness of the antidepressant treatment. Of the patients who considered themselves successfully treated, 24% (124/521) and 17% (149/859) still experienced a severe lack of motivation and difficulty in concentration, respectively.

**Table 1:** Demographic characteristics and comorbid conditions in the total PatientsLikeMe major depressive disorder community (study 1). Percentages are based on the number of available cases for each question.

Variable		Patients with major depressive disorder (N=17,166)
**Country, n (%)**		
	United States	10,349 (75)
	United Kingdom	1203 (9)
	Canada	701 (5)
	Australia	525 (4)
	Other	948 (7)
Sex (female), n (%)		12,072 (77)
Age (years), mean (SD)		40.5 (13.1)
Age at first diagnosis (years), mean (SD)		28.1 (11.7)
**Race, n (%)**		
	White	6648 (89)
	Black	202 (3)
	Other	642 (9)
	Prefer to skip	N/A^a^
**Education, n (%)**		
	Less than high school	370 (6)
	High school graduate	860 (13)
	Some college	2935 (45)
	Undergraduate degree	1450 (22)
	Graduate degree	860 (13)
	Prefer to skip	N/A^a^
Treatment^b^, n (%)		13,367 (78)
**Current depressed mood severity, n (%)**		
	Mild	1964 (25)
	Moderate/severe	5024 (65)
	None	802 (10)
**Comorbidities, n (%)**		
	Generalized anxiety disorder	7881 (46)
	Panic disorder	4582 (27)
	Posttraumatic stress disorder	4344 (25)
	Persistent depressive disorder (dysthymia)	4139 (24)
	Social anxiety disorder	4116 (24)
	Fibromyalgia	3501 (20)
	Tobacco addiction	2940 (17)
	Obsessive compulsive disorder	2612 (15)
	Eating disorder	2297 (13)
	Attention deficit/hyperactivity disorder	2272 (13)
	Phobic disorder	2089 (12)
	Alcohol addiction	1659 (10)
	Drug addiction	1588 (9)
	Bipolar disorder	1479 (9)

^a^N/A: not applicable (“prefer to skip” was not an option within the PatientsLikeMe platform).

^b^Number of individuals who had ever received treatment (treatment predominantly refers to medication, but also includes nonpharmacologic treatments such as psychotherapy).

#### Use of Study 1 Results for Development of the Study 2 Survey

The study 1 results highlighted three key topics to be included in the study 2 survey questionnaire: patient prioritization of symptoms, patient perception of cognitive symptoms of depression, and patient perception of residual symptoms and medication effectiveness.

Regarding patient prioritization of symptoms, patients reported lack of motivation and problems in concentrating as their most severe symptoms while experiencing a severely depressed mood. For some patients, these suggested mood and cognitive symptoms could be a higher priority for symptom resolution than physical symptoms (eg, fatigue and muscle tension). Therefore, the first key objective for study 2 was to understand how patients prioritize resolution of symptoms and how they define their own depression.

With regard to patient perception of cognitive symptoms of depression, study 1 results indicated that the majority of patients suffered some level of impairment in concentration, even when they reported no or mildly depressed mood. However, no data were available to assess other domains of cognition symptoms, such as executive function, attention, and memory; therefore, the second key objective of study 2 was to understand patient perceptions of cognitive symptoms related to depression, of these symptoms on their disorder, their daily life, and as symptoms independent of mood symptomatology.

Study 1 also suggested that many patients experienced residual symptoms when receiving a treatment that was perceived to be effective, including severe lack of motivation and problems concentrating. However, PatientsLikeMe data do not distinguish between residual symptoms of depression and treatment-related side effects. Thus, study 2 aimed to identify residual symptoms that were most important to patients, expectations of symptom resolution, and factors that influence perception of medication efficacy.

### Study 2

#### Patients

The questionnaire survey sample included 525 members with MDD. Survey respondents had demographic characteristics similar to those of the “total PatientsLikeMe MDD community” (Table 2). As in study 1, the majority of participants were women (74%), white (87%), and living in the United States (95%), and 90% had at least some college education, undergraduate degree, or graduate degree.

**Table 2:** Demographic characteristics and comorbid conditions in major depressive disorder survey participants (study 2). Percentages are based on the number of available cases for each question.

Variable		Major depressive disorder survey participants (N=525)
**Country, n (%)^a^**		
	United States	499 (95)
	United Kingdom	8 (2)
	Canada	10 (2)
	Australia	2 (0)
	Other	6 (1)
Sex (female), n (%)		368 (74)
Age (years), mean (SD)		49.0 (12.9)
Age at first diagnosis (years), mean (SD)		29.8 (12.9)
**Race, n (%)**		
	White	432 (87)
	Black	15 (3)
	Other	42 (8)
	Prefer to skip	9 (2)
**Education, n (%)**		
	Less than high school	7 (1)
	High school graduate	42 (8)
	Some college	217 (44)
	Undergraduate degree	134 (27)
	Graduate degree	92 (18)
	Prefer to skip	6 (1)
PHQ-8^b^ score, mean (SD)		23 (6.0)
**PHQ-8 (score), n (%)**		
	None (0-4)	0 (0)
	Mild (5-9)	9 (2)
	Moderate (10-14)	47 (9)
	Moderately severe (15-19)	103 (20)
	Severe (20-24)	354 (67)
	Missing^c^	12 (2)
Currently taking a medication, n (%)		437 (87)
**Number of lifetime depression episodes, n (%)**		
	1 episode	10 (2)
	2–3 episodes	47 (9)
	4–5 episodes	43 (8)
	>5 episodes	374 (71)
	I don’t know/I prefer to skip	51 (10)
**Current symptom status, n (%)**		
	Current episode <3 months/symptoms not better	60 (11)
	Current episode >3 months/symptoms not better	172 (33)
	Current episode >3 months/symptoms better	127 (24)
	No current episode/occurred in the past	127 (24)
	Other	22 (4)
	I don’t know/I prefer to skip	17 (3)
**Comorbidities^d^** **, n (%)**		
	Generalized anxiety disorder	252 (48)
	Posttraumatic stress disorder	224 (43)
	Panic disorder	138 (26)
	Fibromyalgia	137 (26)
	Persistent depressive disorder (dysthymia)	99 (19)
	Hypertension	69 (13)
	Hypothyroidism	65 (12)
	Traumatic brain injury	62 (12)
	Gastroesophageal reflux disease	61 (12)
	Migraine	57 (11)
	Irritable bowel syndrome	55 (10)
	Bipolar disorder	54 (10)
	Asthma	53 (10)
	Social anxiety disorder	51 (10)
	Osteoarthritis	51 (10)

^a^Values reflect answers reported by patients on their PatientsLikeMe profile. Country of origin was not asked directly in the survey.

^b^PHQ-8: patient health questionnaire (8-item).

^c^In accordance with PHQ-8 scoring guidelines, patients with one or more missing item score did not receive a total score.

^d^Values reflect answers reported by patients on their PatientsLikeMe profile.

The characteristics of survey participants are shown in Table 2. PHQ-8 scores indicated moderate (9%), moderately severe (20%), and severe (67%) rates of depression, with a mean score of 23 (SD 6.0). A total of 44% of participants reported a current episode with continued symptoms. In addition, 71% of patients reported at least five past depressive episodes, and 87% reported currently taking medications for MDD. In total, according to data from the PatientsLikeMe platform database, 79% of the survey participants (416/525) reported more than three comorbid conditions—most commonly, generalized anxiety disorder, posttraumatic stress disorder, panic disorder, and dysthymia. The most-common nonpsychological conditions were fibromyalgia, hypertension, hypothyroidism, and gastroesophageal reflux disease.

#### Perception of Symptoms

When patients were asked whether they were able to tell if individual symptoms were improving, 73% of patients (383/525) agreed that they could distinguish between different symptoms of their depression and discern improvements in individual symptoms. In addition, although 72% of respondents (364/508) stated that the symptoms they most wanted to “go away or control” were mood and physical symptoms (depressed mood, anhedonia, anxiety, fatigue, insomnia, muscle tension, feelings of guilt or inadequacy, and low self-esteem), 23% (118/508) cited that the most important symptoms were cognitive symptoms (difficulty staying motivated, difficulty staying focused on goals, concentration problems, short-term memory problems, inability to make decisions, and attention problems).

#### Effects of Cognitive Symptoms on Daily Functioning

Overall, 83% of respondents (433/523) reported speaking with their doctor about cognitive problems, and when asked to describe the experience (via free-text responses), 73% of the respondents (172/237) reported that they had received advice or treatment. The mean PDQ-5 score of respondents was 13.2 (SD 4.2), with 31% of respondents (165/525) scoring a higher level of perceived cognitive impairment (scores of 16-20).

Most patients attributed their cognitive difficulties to a combination of factors, including MDD (82%, 432/524) and other health (eg, fibromyalgia and Parkinson disease; 74%, 390/524) and life (eg, stress; 84%, 439/524) factors; only 6% (30/524) attributed these symptoms solely to MDD.

**Figure 3:**
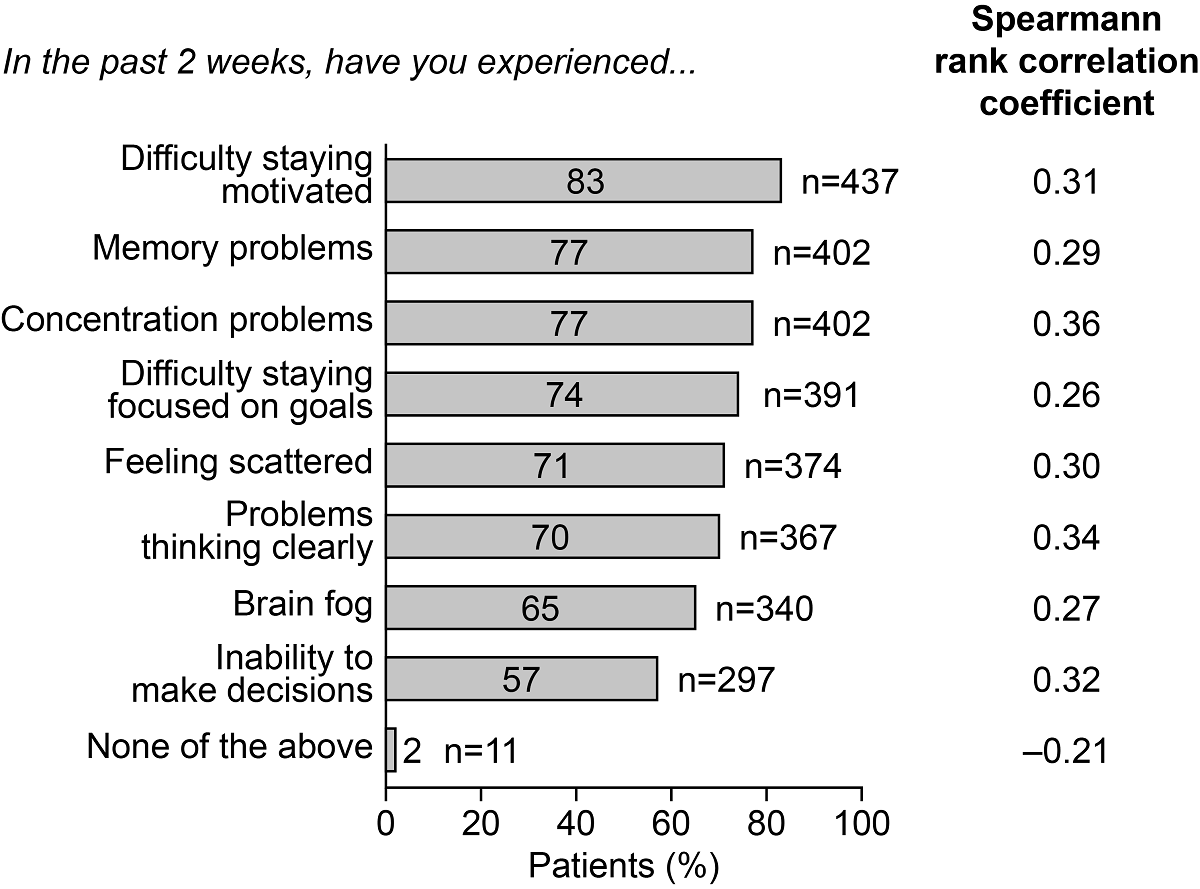
Self-reported cognitive symptoms among survey respondents with major depressive disorder in study 2, and correlation with severity of depression (N=525).

Many patients reported cognitive symptoms other than those recorded in the PDQ-5. The cognitive symptoms that were most strongly correlated with severity of depression, as measured by the PHQ-8, were difficulty making decisions, concentrating, and thinking clearly (Spearman rank correlation coefficients [r_s_] of 0.32, 0.36, and 0.34, respectively; Figure 3).

**Figure 4:**
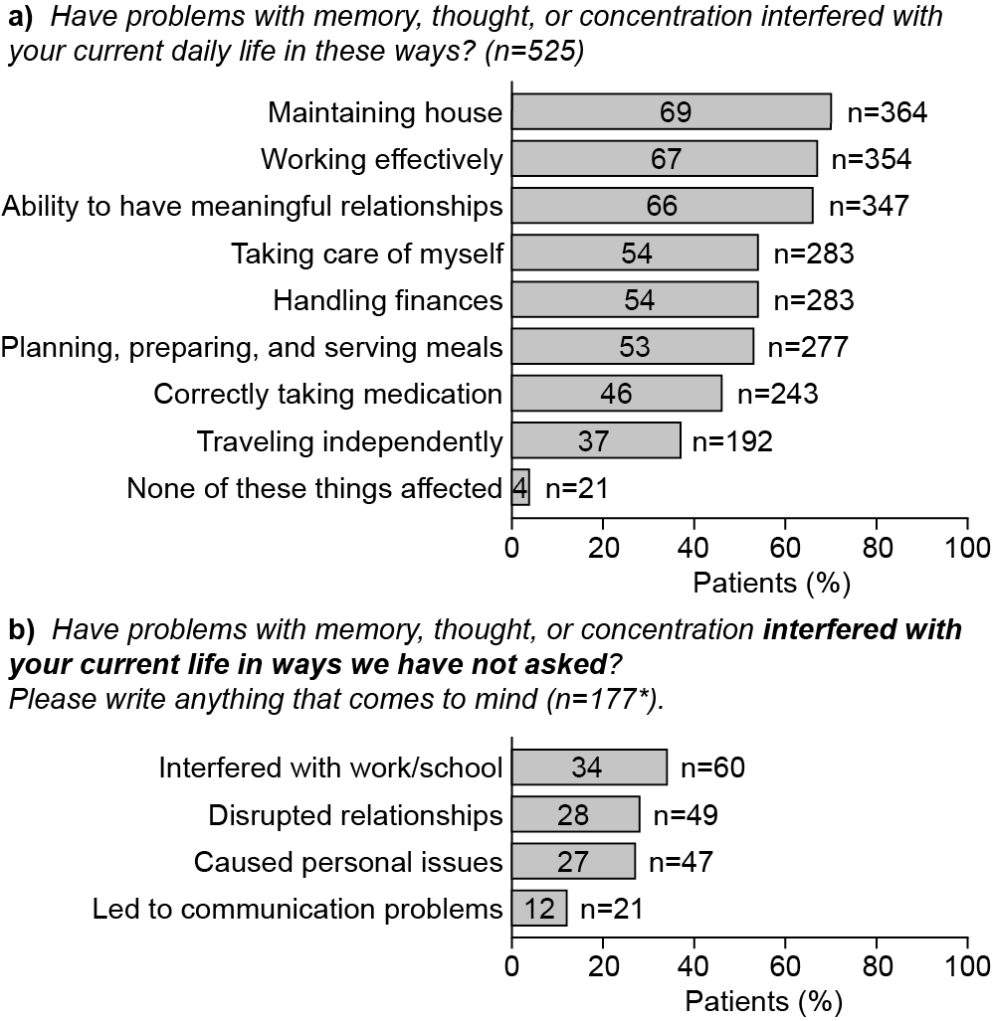
Effects of cognitive symptoms on social functioning among survey respondents in study 2. *Categories are not exclusive; a category is counted each time it is mentioned by the respondent.

When asked to select three symptoms with the most negative impact, depressed mood was the most frequently reported symptom to have a negative impact on family and social relationships (39%, 196/509) and fatigue had the most negative impact on social and leisure activities (43%, 219/510). Problems with memory, motivation, and concentration/attention also interfered with these aspects of life. In all, 66% of respondents stated that cognitive difficulties interfered with their ability to have meaningful relationships, and 69% reported that cognitive difficulties impaired their ability to maintain the household (Figure 4). More than half the patients reported that memory, thought, and concentration difficulties reduced their ability to handle household finances (54%), plan and serve meals (53%), and take care of themselves (54%; Figure 4a). When asked to provide free-text responses about how cognitive symptoms impacted them in any way that was not previously asked, common response themes among the respondents included interference with work/school (34%), disrupted relationships (28%), personal issues (27%), and communication problems (12%; Figure 4a).

Cognitive difficulties also had a profoundly negative impact on patients’ ability to work. In all, 35% of respondents cited poor concentration as one of the three symptoms with the most negative impact on occupational ability, compared with only 22% who cited symptoms of depressed mood (Multimedia Appendix 2). In total, 65% of respondents (341/525) reported that memory, thought, and concentration difficulties interfered with their ability to work effectively. A total of 48% of patients had stopped working completely as a result of the disability caused by these symptoms, and only a minority were employed full time (18%, 88/498) or part time (8%, 41/498).

In the free-text responses, patients commonly used the terms “brain fog,” “memory,” and “remember” when describing their cognitive symptoms. Free-text analysis revealed themes of feeling paralyzed, debilitated, and frustrated by symptoms:

When I am unable to concentrate I do not do anything well. It affects my job performance...I fall behind in all the things I need to do at work and in my personal life which triggers more depression and other negative emotions.

...being indecisive is like mental paralysis.

I feel like other symptoms would be easier to manage if I could stay focused.

#### Cognition and Recovery From Major Depressive Disorder

Patients reported that cognitive symptoms “often” or “almost always” interfered with their ability to recover fully from depression (24% [125/525] and 19% [98/525] respondents, respectively), prevent relapse (22% [116/525] and 16% [84/525], respectively), and participate actively in treatment (15% [78/525] and 8% [43/525], respectively). Therefore, patients recognized cognitive difficulties as a distinct entity from other symptoms of depression.

Most patients reported multiple lifestyle adjustments to try to overcome the challenges posed by their cognitive symptoms. These included using reminders (eg, electronic organizer, checklists, and sticky notes; 85%, 439/516), organizing their living space (49%, 255/516), and taking public transportation instead of driving themselves (15%, 79/516). Patients stated that relief from emotional symptoms (91%; 454/501 respondents) and relief from cognitive symptoms (75%, 378/501) were “very important” in treatment decisions; in addition, a number of patients reported relief from emotional symptoms (7%, 37/501) and cognitive symptoms (19%, 96/501) as “somewhat important.” However, 61% of respondents (319/525) considered antidepressant treatment successful as long as their most troublesome symptoms improved. Overall, 9% of the respondents (46/501) stated that “the need to think more clearly” was the main priority in selecting antidepressant treatment, compared with 17% (85/501) who cited low rates of side effects and 13% (64/501) who were guided primarily by HCP recommendations.

## Discussion

### Principal Findings

Our study results suggest that patients with MDD recognize improvement in individual symptoms of depression and can discern how changes in different symptom domains affect various aspects of their lives. Although many patients were aware of their cognitive difficulties, not all patients attributed them to MDD.

Although most participants in the PIVOT survey (study 2) reported improvement in mood and physical symptoms as their primary treatment goal, almost a quarter prioritized cognitive symptoms as those they most wanted to “go away or control.” However, 61% of respondents in study 2 also stated that they would consider antidepressant treatments successful if they saw improvements in their most troublesome symptoms. This finding may be indicative of a discrepancy between the high prioritization of resolution of cognitive symptoms by patients and the perception that their treatment is working even when these symptoms persist. Therefore, both HCPs and patients need to know that cognitive symptoms are a core feature of MDD and that successful treatment of MDD should equate to not only improvements in mood, but also to recovery from all aspects of the disorder. HCPs should be aware of the link between residual symptoms (including cognitive symptoms) and the increased risk of relapse [12]. Moreover, it may be beneficial for HCPs to further address medical comorbidities in MDD, such as sleep deprivation, which are known to negatively affect cognitive performance and might contribute to improved patient outcomes [10].

Although the majority of respondents in study 2 had talked to their HCP about cognitive problems at some point and many received advice or treatment, patients reported that cognitive difficulties persisted. On the PDQ-5, a measure of perceived cognitive dysfunction severity, 31% of patients scored in the highest quintile, with a mean PDQ score of 13.2 (SD 4.2). These patient-reported findings support the empirical evidence, suggesting that although conventional antidepressants mitigate deficits in certain cognitive domains in a proportion of patients [8], outstanding deficits in executive functioning [27-29] often persist even after the depressive symptoms have remitted [30-33]. However, evidence for which cognitive domains remain impaired after antidepressant treatment is inconsistent [27,29,34]. This inconsistent finding emphasizes that identification and treatment of cognitive symptoms are important unmet clinical needs among patients with MDD, including those whose depressive symptoms have remitted, and highlights the need for HCP and patient education on identification, treatment, and follow-up of cognitive symptoms in MDD.

In the PIVOT studies, cognitive difficulties had far-reaching effects on multiple aspects of patients’ lives. Patients recognized that cognitive symptoms, distinct from depressed mood, disrupted their relationships, ability to carry out household duties, and ability to hold down a job or work full-time. Patient-reported data from the present study support those from a post hoc analysis from the International Mood Disorders Collaborative Project, which reported that workplace performance variability is explained by subjective measures of cognitive symptoms to a greater extent than by total depression symptom severity [35].

A notable proportion of respondents indicated that cognitive symptoms interfered with work ability; therefore, it would be valuable to increase awareness about the effects of cognitive difficulties on work ability, even following remission of depressive symptoms, among HCPs and payers. The well-being of patients with cognitive symptoms in MDD should be considered by providing workplace interventions, including work modification and support programs, exposure-based work reintegration plans, and problem-solving therapy. Such interventions, when implemented alongside clinical intervention, were found to reduce sick leave among employees with depressive disorders in the medium term (4 to 12 months) [36].

Beyond functioning at work, the potential negative effects of cognitive dysfunction on social and family relationships cannot be minimized. At least two-thirds of respondents reported difficulties in maintaining meaningful relationships, but other limitations may have indirect effects on relationships. For example, more than half of the respondents reported difficulties maintaining the household, managing finances, taking care of oneself, and preparing meals. One would expect that these difficulties place additional burden on caregivers. Because these activities involve attention, planning, and decision making, cognitive functions such as executive functioning, attention capacity, and memory have been implicated [37]. However, additional research is needed to determine the causal pathway between cognitive dysfunction and psychosocial deficits in major depression.

Many patients reported that cognitive symptoms “often” or “almost always” interfered with their ability to fully recover from depression; thus, HCPs need to be able to evaluate the potential of residual cognitive symptoms to trigger future relapses of MDD and be aware of the more sensitive assessment of cognition at all phases of the depression cycle. Overall, a marked proportion of patients cited relief from cognitive symptoms as an important factor when deciding on a treatment for MDD. This finding implies that treatments for MDD that improve cognitive functioning may be preferred by patients over options that do not address this aspect of the disorder. Several new or repurposed medications that target cognitive symptoms may help address this need, including vortioxetine, lisdexamfetamine, and erythropoietin [31]; however, the long-term benefits of such MDD treatments in cognitive function remain to be fully elucidated [38].

Overall, HCPs may want to adopt a rehabilitation approach for the assessment and treatment of cognitive symptoms in MDD and utilize cognitive remediation in addition to occupational therapies. Since cognitive symptoms are increasingly recognized as a factor that can impair recovery from MDD and maintain psychosocial and workplace disability, even after MDD remittance [9,33], some experts recommend that “cognitive remission” and “functional recovery” be added as treatment goals for MDD [39,40].

### Limitations

There are some limitations to this study that should be noted. The majority of participants in this study were white, educated women from the United States who had insurance coverage, which is higher than the rates of women and white patients reported in a previous national study of depressive symptoms (58.1% women and 73.4% white patients) [41]. This inequality in demographics may therefore limit generalizability. However, it should also be noted that some attributes such as the mean age and age at first episode were similar to those reported in previous studies [42]. In addition, owing to the self-selected nature of the PatientsLikeMe patient population and the lack of an independent validation of diagnosis and cognitive impairment, it is not possible to confirm that patients represented in the PatientsLikeMe platform and survey data were indeed diagnosed with MDD. However, participants who participated in the survey completed the PHQ-8 depression scale, and almost 87% were found to have moderately severe or severe levels of depression. Finally, the study design did not allow an independent assessment to determine whether participants were experiencing actual cognitive performance difficulties. Previous studies have found that subjective impressions and objective performance in cognition do not correlate with each other [43] and may reflect the negative cognitive bias central to MDD [10]. Therefore, it is possible that patients’ self-reports may not have mirrored their cognitive reality. Moreover, previous studies have found that subjective reports of cognitive dysfunction correlate with depression symptoms [43].

Further studies are needed to evaluate the consequences of residual cognitive symptoms after remission of depression and identify appropriate treatments. It is important for future studies to evaluate patient perceptions of cognitive function along with objective longitudinal measurement through the cycle of depression and to investigate the effect of medications and cognitive remediation on the course and treatment outcomes of depression.

### Conclusions

The PIVOT studies provide an understanding of how symptoms of and recovery from MDD are conceptualized by patients. An analysis of the PatientsLikeMe database responses indicated that cognitive symptoms were frequently reported, and many patients with no/mild depression still reported difficulty in concentrating. These difficulties were also reported among patients who considered their treatment successful. In the prospective survey, most patients reported the ability to discern improvements in individual MDD symptoms. Cognitive symptoms correlated with depression severity, and patients acknowledged that cognitive symptoms did not necessarily improve with treatment. These symptoms interfered with meaningful relationships and daily life tasks and impacted work and recovery from depression. Our studies identified the need for measures to further characterize the nature and impact of cognitive symptoms in MDD and a commitment to integrate cognitive function into the assessment and management of MDD. Further understanding and management of cognitive issues of MDD will be beneficial for patients and may improve overall recovery.

## Appendix

Multimedia Appendix 1 Full PIVOT study survey. PIVOT: Patient Insights and Voice on Major Depressive Disorder Treatment and Symptom Perception.

Multimedia Appendix 2 Effect of cognitive and mood disturbance symptoms on ability to work among survey respondents in study 2.

## Abbreviations

HCP: health care providers
MDD: major depressive disorder
PDQ: perceived deficits questionnaire
PHQ: patient health questionnaire
PIVOT: Patient Insights and Voice on Major Depressive Disorder Treatment and Symptom Perception
