# Supplementary material for: Patient Perspective of Cognitive Symptoms in Major Depressive Disorder: Retrospective Database and Prospective Survey Analyses
Source: J Particip Med. 2019 May 16;11(2):e11167. doi: 10.2196/11167 (PMC7434060; doi:10.2196/11167)
Supplement: Multimedia Appendix 1 [file jopm_v11i2e11167_app1.pdf]

Multimedia Appendix 1

Section 1:

[3 Questions] Welcome to the **PIVOT** Study (**P**atient **I**nsights and **V**oice **o**n MDD **T**reatment Efficacy and Symptom Perception). This survey is part of our research collaboration between PatientsLikeMe and Takeda/Lundbeck. We appreciate your time and willingness to share your experiences with us. The goal of this study is to better understand your symptoms of major depressive disorder and which ones are most troublesome and hardest to resolve. During this study we will ask a variety of questions related to your experience with depression.

First we would like to know a little more about your experience living with Major Depressive Disorder (MDD). This helps us understand where in your journey you are and enables us to compare MDD member experiences.

1. How old were you when you were first diagnosed with Major Depressive Disorder? Please enter a whole number.

   [age]

2. During your life, how many major depression episodes have you had? These can be defined as depression followed by recovery or remission of the major depression episode.

1. 1 episode
2. 2-3 episodes
3. 3-5 episodes
4. More than 5 episodes
5. I don’t know

1. We would like you to think about your depression **today**. Which of the following most accurately describes your current depression? We understand these may not completely describe your situation. Please choose the **best** answer.

1. My current depressive episode started less than 3 months ago and I have a lot of symptoms.
2. I am currently in a depressive episode that has lasted **more than 3 months** and I have a lot of symptoms.
3. I am currently in a depressive episode that has lasted **more than 3 months** and many or most of my symptoms have gotten better.

4. I do not consider myself to be currently in a depressive episode, although I have had one or more in the past.
5. Other [fill in blank]
6. I don’t know

Section 2: Current Symptoms of Depression *[this is the Patient Health Questionnaire Depression Scale (PHQ-8)]*

[8 questions]. Next we are going to ask you about your current experience of Major Depressive Disorder. Over the **last 2 weeks**, how often have you been bothered by any of the following problems?

	Not at all	Several days	More than half the days	Nearly every day
Little interest or pleasure in doing things				
Feeling down, depressed, or hopeless				
Trouble falling or staying asleep, or sleeping too much				
Feeling tired or having little energy				
Poor appetite or overeating				
Feeling bad about yourself, or that you are a failure, or have let yourself or your family down				
Trouble concentrating on things, such as reading the newspaper or watching television				
Moving or speaking so slowly that other people could have noticed. Or the opposite - being so fidgety or restless that you have been moving around a lot more than usual				

Section 3: Experiences Related to Memory, Attention, and Concentration - Part 1

[7 Questions] The next set of questions will further explore your experience with MDD.

Many people with Major Depressive Disorder (MDD) have problems with thinking, memory, and concentrating. For some, once the depression is treated, the “brain fog”, feelings of being “scattered”, or being unable to concentrate gets better too. But, for some people with MDD, the problems with thinking continue to be troublesome.

1. In the past 2 weeks, which of the following have you experienced?
   Please check all that apply.
   1. Memory problems
   2. Thinking problems
   3. Problems concentrating
   4. Brain fog
   5. Feeling “scattered”
   6. Problems staying motivated
   7. Inability to make decisions
   8. Difficulty staying focused on goals
   9. None of the above (go to Question Q1B, all other answers go to Q2)

   1B. Which of the following have you **previously experienced** due to major depressive disorder?
   Please check all that apply.
   1. Memory problems
   2. Thinking problems
   3. Problems concentrating
   4. Brain fog
   5. Feeling “scattered”
   6. Problems staying motivated
   7. Inability to make decisions
   8. Difficulty staying focused on goals (a-h go to Q2)
   9. None of the above (skip to next section)

2. What words have you used to describe your experience of having problems with your memory, thinking, or concentration? Please write anything that comes to mind.

   [OPEN TEXT]

3. You said you experience difficulties with [first response given in Q1]. Do you feel your difficulties with [first response given in Q1] are due to:
   1. Due to major depressive disorder (MDD) (skip to Q5)
   2. Due to MDD and other health condition
   3. Due to other health condition
   4. I’m not sure (skip to Q5)

4. What is this health condition?
   [OPEN TEXT/auto complete]

5. Have problems with your memory, thinking, or concentration interfered with your **current** daily life in these ways? Please select all that apply.

1. Ability to handle finances
2. Ability to take medication in correct dosages at the correct time
3. Ability to take care of myself
4. Ability to travel independently on public transportation or drive own car

5. Ability to maintain house (e.g. daily tasks like dishwashing, bed-making, cleanliness)
6. Ability to plan, prepare and serve adequate meals independently
7. Ability to work effectively
8. Ability to have meaningful relationships with friends and/or family
9. None of these things have been affected

6. Have you had to change your job or career path based on problems with memory, thinking, or concentration?

1. Yes, I had to stop working completely
2. Yes, I had to make changes at my job (e.g. work less hours)
3. Yes, I had to switch jobs
4. No, I haven't had to change my job or career path

7. Have problems with your memory, thinking, or concentration interfered with your **current** life in ways we have not asked? Please write anything that comes to mind.

[OPEN TEXT]

Section 4: Experiences Related to Memory, Attention, and Concentration - Part 2

[*The first section is the Perceived Deficits Questionnaire-5 (PDQ) which measures one symptom in each cognitive domain]*

[11 questions] Everyone at some point experiences problems with memory, attention, or concentration, but these problems may occur more frequently for individuals with Major Depression Disorder. The following questions describe several situations in which a person may encounter problems with **memory, attention, or concentration**.

Please select the appropriate response based on your cognitive function during the **past 4 weeks**. Please answer every question. If you are not sure which answer to select, please choose the one that comes closest to describing you.

During the past 4 weeks, how often did you...

	Never	Rarely	Sometimes	Often	Almost Always
Have trouble getting things					

organized?					
Have trouble concentrating on things like watching a television program or reading a book?					
Forget the date unless you looked it up?					
Forget what you talked about after a telephone conversation?					
Feel like your mind went totally blank?					

1. To what extent do you experience problems in thinking (such as memory, attention span, planning, learning new skills) that occur no matter what your mood is?
    1. Almost all of the time
    2. Often
    3. Sometimes
    4. Rarely
    5. Never

Next we want you to think about how your ability to **think clearly** (such as memory, attention span, planning, learning new skills) **interferes with your ability** to do certain things.

To what extent do you think your ability to think clearly interferes with your ability to:

	Never	Rarely	Sometimes	Often	Almost Always
Participate actively in treatment					
Follow treatment recommendations					
Make decisions that help you deal with your depression					
Recover fully from your depression					
Prevent depression from coming back					

Section 5: Seeking advice and life adjustments [4 questions]

1. Have you ever spoken to your doctor about difficulties with your memory, thinking, or concentration?
1. Yes
2. No
3. Do not want to answer

2. What was that experience like? Did your doctor offer advice on treatment or managing your symptoms?

[OPEN TEXT]

3. Are there adjustments in your life that you have made to overcome problems or to improve your memory, thinking, or concentration?
1. Organize your living space and/or work space (Examples: Have a central location to keep your keys; using a notepad system beside the phone)
2. Use memory aids (Examples: Make checklists, use timers or electronic organizer, use Post-it notes)
3. Focus on one task at a time and fully complete it, before moving on to the next task
4. “Train” your brain (crossword puzzles, sudoku, logic puzzles, games, etc.)
5. Taken public transportation or reduced time of continuous driving
6. I don’t know

4. Are there other adjustments in your life that you have made or anything else you would like to tell us about?

[OPEN TEXT]

Section 6:

[7 questions] Often people with Major Depressive Disorder experience several different kinds of emotional, physical, and thinking problems. Common symptoms may include depressed mood, anxiety, inability to experience pleasure, fatigue, problems concentrating, among others. But, symptoms of depression can be very specific to each person. We would like you to think about your **current experience** of depression in this section.

1. **Currently,** what symptom(s) are most severe?

Please choose up to 3:

1. Depressed mood
2. Inability to experience pleasure (anhedonia)
3. Anxiety
4. Fatigue
5. Insomnia
6. Muscle tension
7. Inability to stay motivated
8. Problems staying focused on my goals
9. Problems concentrating
10. Problems with my short-term memory
11. Problems making decisions
12. Attention problems
13. Feelings of guilt or inadequacy
14. Low self-esteem
15. Other [text]
16. None

2. **Currently,** what symptom(s) do you find the most **bothersome**?

Please choose up to 3:

1. Depressed mood
2. Inability to experience pleasure (anhedonia)
3. Anxiety
4. Fatigue
5. Insomnia
6. Muscle tension
7. Inability to stay motivated
8. Problems staying focused on my goals
9. Problems concentrating
10. Problems with my short-term memory
11. Problems making decisions
12. Attention problems
13. Feelings of guilt or inadequacy
14. Low self-esteem
15. Other [text]
16. None

3. **Currently,** what symptom(s) has had the most negative impact on your **ability to work**? **Please choose up to 3:**

1. Depressed mood
2. Inability to experience pleasure (anhedonia)
3. Anxiety
4. Fatigue
5. Insomnia
6. Muscle tension
7. Inability to stay motivated
8. Problems staying focused on my goals
9. Problems concentrating
10. Problems with my short-term memory
11. Problems making decisions
12. Attention problems
13. Feelings of guilt or inadequacy
14. Low self-esteem
15. Other [text]
16. None

4. **Currently,** what symptom(s) has had the most negative impact on your **social and leisure activities**? **Please choose up to 3:**

1. Depressed mood
2. Inability to experience pleasure (anhedonia)
3. Anxiety
4. Fatigue
5. Insomnia
6. Muscle tension
7. Inability to stay motivated
8. Problems staying focused on my goals
9. Problems concentrating
10. Problems with my short-term memory
11. Problems making decisions
12. Attention problems
13. Feelings of guilt or inadequacy
14. Low self-esteem
15. Other [text]
16. None

5. **Currently,** what symptom(s) has had the most negative impact on your **family or partner/spouse relationships**? **Please choose up to 3:**

1. Depressed mood
2. Inability to experience pleasure (anhedonia)
3. Anxiety
4. Fatigue
5. Insomnia
6. Muscle tension
7. Inability to stay motivated
8. Problems staying focused on my goals
9. Problems concentrating
10. Problems with my short-term memory
11. Problems making decisions
12. Attention problems
13. Feelings of guilt or inadequacy
14. Low self-esteem
15. Other [text]
16. None

6. **Currently,** what **one symptom** related to your MDD do you most want to **go away or control**? **Please choose one:**

1. Depressed mood
2. Inability to experience pleasure (anhedonia)
3. Anxiety
4. Fatigue
5. Insomnia
6. Muscle tension
7. Inability to stay motivated
8. Problems staying focused on my goals
9. Problems concentrating

10. Problems with my short-term memory
11. Problems making decisions
12. Attention problems
13. Feelings of guilt or inadequacy
14. Low self-esteem
15. Other [text]
16. None

7. What is it about this symptom that makes it the most important to go away or control?

[OPEN TEXT]

Section 7: Medications for Major Depressive Disorder [3 questions]

Next we will briefly ask about medications you may have taken to treat your depression.

1. Please choose one answer for each medication.

Medication	Yes, I am **currently** taking this medication	Yes, I have tried this but I am **no longer** taking this medication	No, I have **not** tried this medication	I am **not sure** if I have tried this medication
Sertraline (Zoloft)				
Fluoxetine (Prozac)				
Duloxetine (Cymbalta)				
Bupropion (Wellbutrin)				
Venlafaxine (Effexor)				
Citalopram (Celexa)				
Escitalopram (Lexapro)				

Vortioxetine<br><br>(Brintellix)				

1. Are you satisfied with your current medication(s) to treat your Major Depression Disorder?
1. Very satisfied
2. Moderately satisfied
3. Neither satisfied nor dissatisfied
4. Moderately dissatisfied
5. Very dissatisfied

2. If you would you consider changing your medications, why might you to consider a different

   treatment? Please select all that apply:
1. Doctor recommendation
2. Fewer side effects (examples: less weight gain, less insomnia)
3. Less expensive medication
4. Relief from an emotional symptoms of depression (examples: depressed mood, anxiety, lack of

   pleasure)
5. Relief from a physical symptom of depression (example: muscle tension)
6. Relief from a cognitive symptom of depression (examples: memory problems, problems concentrating

   or thinking)
7. I wouldn’t consider changing my medications at this time
8. Other reasons [OPEN TEXT]

Section 9: Demographic and Patient Characteristics Module [5 questions]:

1. Are you...
1. Male
2. Female
3. I prefer not to say

2. What is your age in full years?

3. What race/ethnicity do you consider yourself to be?
1. White
2. Black or African-American
3. Asian
4. Hispanic or Latino
5. Native Hawaiian or other Pacific Islander
6. American Indian or Alaskan Native
7. Mixed race
8. I prefer not to say

4. What is your highest level of education?
1. Less than high school graduate
2. High school graduate or GED
3. Some college but less than an associate’s degree/ bachelor’s degree
4. Bachelor’s or Associate’s degree
5. Postgraduate degree (Master's, doctorate, law, medical or equivalent)
6. I prefer not to say

5. Which of the following **best** describes your employment status? Please choose only one:
1. Employed, full time
2. Employed, part time
3. Student
4. Retired
5. Unemployed and looking for work
6. Unemployed and not looking for work
7. Disabled/Unable to work
8. Other
9. I prefer not to say.

****

(End of survey)
