# Supplementary material for: Patient Perspective of Cognitive Symptoms in Major Depressive Disorder: Retrospective Database and Prospective Survey Analyses
Source: J Particip Med. 2019 May 16;11(2):e11167. doi: 10.2196/11167 (PMC7434060; doi:10.2196/11167)

Supplement for:

The Patient Perspective on Cognitive Symptoms in Major Depressive Disorder: A Retrospective Database and Prospective Survey Analysis

E Chiauzzi, J Drahos, S Sarkey, C Curran, V Wang, D Tomori

Supplementary text Study 2 prospective survey questionnaire

Supplementary Figure 1. Effect of cognitive and mood disturbance symptoms on ability to work among survey respondents in study 2.

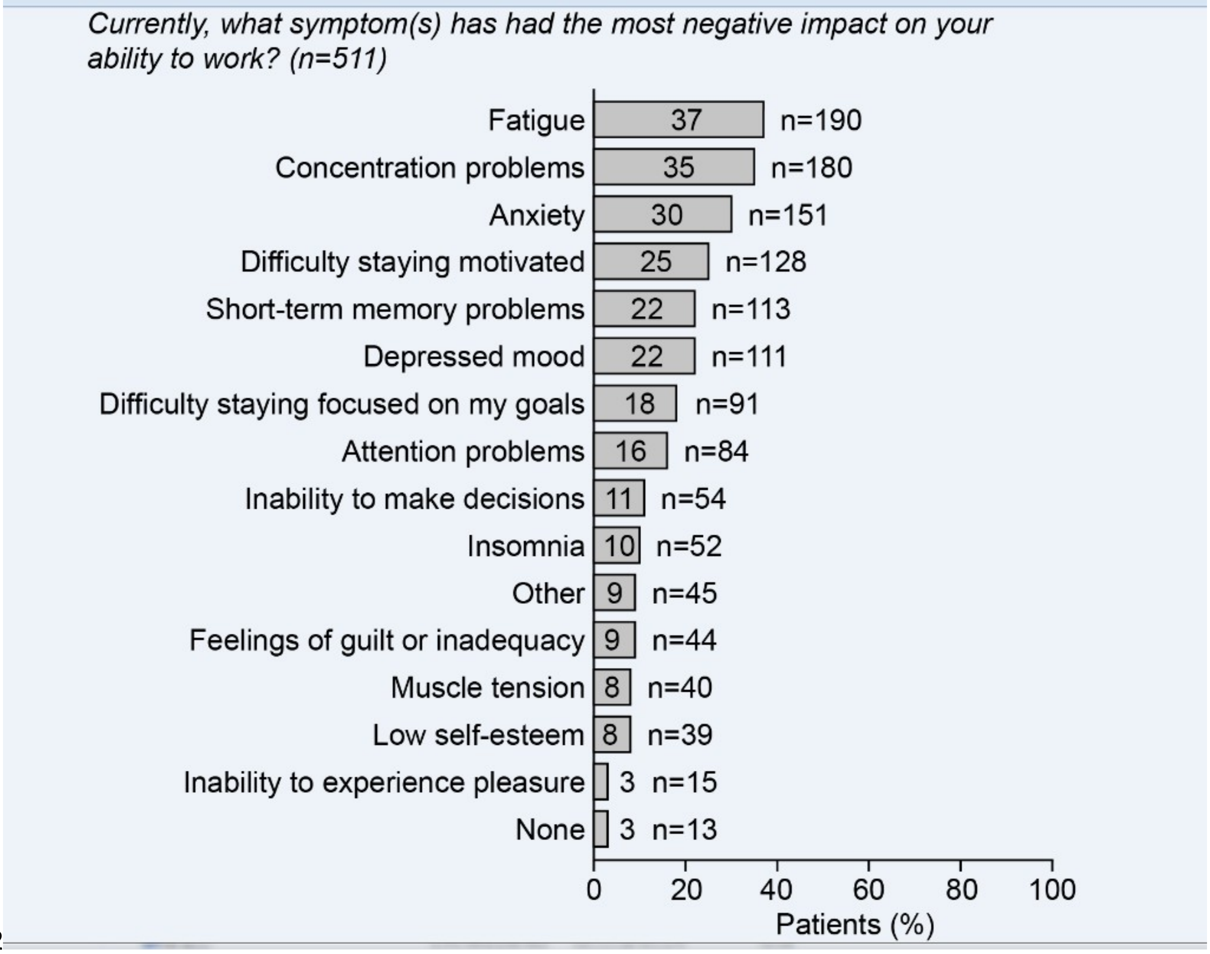

Supplement: Multimedia Appendix 2 [file jopm_v11i2e11167_app2.pdf]
